# Current Perspectives on Viable but Non-culturable State in Foodborne Pathogens

**DOI:** 10.3389/fmicb.2017.00580

**Published:** 2017-04-04

**Authors:** Xihong Zhao, Junliang Zhong, Caijiao Wei, Chii-Wann Lin, Tian Ding

**Affiliations:** ^1^Key Laboratory for Green Chemical Process of Ministry of Education, Key Laboratory for Hubei Novel Reactor and Green Chemical Technology, School of Chemical Engineering and Pharmacy, Wuhan Institute of TechnologyWuhan, China; ^2^Institute of Biomedical Engineering, National Taiwan UniversityTaipei, Taiwan; ^3^Department of Food Science and Nutrition, Zhejiang Key Laboratory for Agro-Food Processing, Zhejiang UniversityHangzhou, China

**Keywords:** VBNC, foodborne pathogens, induction, detection method, resuscitation

## Abstract

The viable but non-culturable (VBNC) state, a unique state in which a number of bacteria respond to adverse circumstances, was first discovered in 1982. Unfortunately, it has been reported that many foodborne pathogens can be induced to enter the VBNC state by the limiting environmental conditions during food processing and preservation, such as extreme temperatures, drying, irradiation, pulsed electric field, and high pressure stress, as well as the addition of preservatives and disinfectants. After entering the VBNC state, foodborne pathogens will introduce a serious crisis to food safety and public health because they cannot be detected using conventional plate counting techniques. This review provides an overview of the various features of the VBNC state, including the biological characteristics, induction and resuscitation factors, formation and resuscitation mechanisms, detection methods, and relationship to food safety.

## Introduction

The viable but non-culturable (VBNC) state, a special physiological state, was first discovered and presented by Xu et al. (1982). As bacteria are subjected to some environmental stress, they cannot grow on conventional culture medium and maintain their activity. Another bacterial non-culturable state that is similar to the VBNC state is dormancy, which is defined operationally as a reversible state of metabolic shutdown (Kell et al., 1998). The VBNC state is presented slightly differently compared with dormancy because VBNC cells exhibit measurable metabolic activity, which is not detected in dormant cells (Mukamolova et al., 2003). However, many authors consider the VBNC state and dormancy as different terms that are used for the same physiological state (Oliver, 2005; Ayrapetyan and Oliver, 2016).

The concept of VBNC has attracted great attention in the fields of microbiology, because it has upset the traditional concept of microorganismal growth. Unlike normal cells, VBNC cells cannot be grown in conventional culture medium, and thus, conventional methods of detection cannot be used to detect bacterial pathogens in the VBNC state. Thus, challenges are encountered in the detection of pathogens. To date, researchers have identified 85 species of bacteria that can enter the VBNC state, including 18 non-pathogenic species and 67 pathogenic species. Some foodborne pathogens retain virulence after entering the VBNC state, which may be due to their rapid resuscitation into culturable cells under certain conditions (Li et al., 2014).

Many people believe that VBNC pathogens are simply in a stage preceding cell death or adaptation to stress (Sachidanandham and Gin, 2009), which cannot induce disease despite the retention of virulent properties. VBNC pathogens are generally considered to be unable to initiate disease, but the virulence of VBNC pathogens can be recovered or maintained after resuscitation, leading to disease/infection (Du et al., 2007b; Nicolò and Guglielmino, 2012). For example, the VBNC cells of *Listeria monocytogenes* resuscitated by incubation with embryonated egg regained virulence identical to that of culturable cells (Cappelier et al., 2007). More seriously, large amounts of evidence have shown that VBNC pathogens may be involved in foodborne outbreaks. For example, a foodborne outbreak caused by salted salmon roe contaminated with Enterohemorrhagic *Escherichia coli* O157 was reported in Japan. Makino et al. (2000) performed numerous experiments and proposed that *E. coli* O157 might enter the VBNC state in salted salmon roe. In another outbreak in Japan, Asakura et al. (2002) suggested that *Salmonella Oranienburg* might become VBNC cells in response to NaCl stress in the outbreak caused by dried processed squids, and this hypothesis was confirmed by resuscitation experiments. Although there is no evidence to confirm that these outbreaks were directly caused by VBNC pathogens, the above studies adequately demonstrate that the potential presence of VBNC pathogens can pose a serious risk to food safety and public health. Foodborne pathogenic bacteria that use food as a carrier are some of the most important human pathogens, causing foodborne disease in human beings (Zhao et al., 2014, 2016). The foodborne pathogens may enter the VBNC state during food processing techniques, such as high temperature, high pressure, disinfectant, preservation, and low temperature storage, and they have become a potential risk for food safety. The VBNC cells of foodborne pathogenic bacteria are easily missed using the conventional plate counting technique and can be recovered with pathogenicity under certain conditions, resulting in a serious threat to human health. Therefore, research investigating VBNC foodborne pathogens is very important, and the establishment of a rapid and effective detection method for bacteria in the VBNC state has become a key to resolving the current crisis, as well as guaranteeing food safety and human health. To provide references for the safety control of foodborne pathogens, the biological characteristics, induction and resuscitation factors, detection methods and formation mechanism of VBNC foodborne pathogens are reviewed in this article.

## Foodborne Pathogens in the VBNC State

After the discovery and presentation of VBNC cells in 1982, researchers found different species of bacteria that can exist in a VBNC state in recent years. For example, Oliver reviewed 52 species of VBNC bacteria in 2005 (Oliver, 2005), and added four new species in his review of pathogenic bacteria published 5 years later (Oliver, 2010). Moreover, 51 species of human pathogens have been reported to exist in the VBNC state (Li et al., 2014). Recently, Pinto et al. (2015) presented a list of 68 species of pathogenic bacteria in the VBNC state which was described. An increasing number of VBNC cells have been found in various environments, which cannot be ignored because of their negative impact on public health. Food is frequently exposed to a limited environment during processing, transportation and storage, which can provide more opportunities for the induction of VBNC cells. Unfortunately, it has been widely documented that foodborne pathogens are induced to enter the VBNC state in various foods, such as grapefruit juice (Nicolò et al., 2010), milk products (Gunasekera et al., 2002; Barron and Forsythe, 2007), and vegetables (Dinu and Bach, 2011, 2013). Additionally, most foodborne pathogens can be induced to enter the VBNC state in response to environmental stress. For example, *E. coli* O157:H7 VBNC cells were induced by low temperature on the surface of lettuce and spinach plants (Dinu and Bach, 2013) and by UV disinfection (Zhang et al., 2015). *Campylobacter jejuni* VBNC cells were observed under oxygen-rich (Oh et al., 2015) and low temperature conditions (Chaisowwong et al., 2012). The cells of *L. monocytogenes* entered a VBNC state within 24 h in the presence of potassium sorbate at pH 4.0 (Cunningham et al., 2009). *L. monocytogenes* and *Bacillus cereus* were also changed into VBNC cells by treatment with a pulsed electric field (Rowan, 2004). The above examples fully illustrate that food samples cannot be considered free from pathogens if the plate counting result is negative. Therefore, an understanding of the VBNC state is essential to comprehend the challenges associated with and how to avoid the risk of VBNC pathogens.

In this review, we specifically focus on foodborne pathogenic bacteria that can enter VBNC state. An overview of 35 foodborne pathogens with a confirmed VBNC state is provided in **Table 1**, with their main survival environments and VBNC cells induction and resuscitation conditions. The list includes many pathogens, such as *E. coli* O157:H7, *Staphylococcus aureus* and *C. jejuni*, which cause foodborne disease in healthy individuals, as observed in outbreaks of epidemic diarrhea or collective food poisoning (Chaisowwong et al., 2012; Dinu and Bach, 2013; Pasquaroli et al., 2014). It also includes some pathogens such as *Vibrio alginolyticus, Vibrio parahaemolyticus*, and *Salmonella* that mainly infect human beings by first infecting other organisms (like poultry, livestock, and seafood) (Bates and Oliver, 2004; Albertini et al., 2006; Braden, 2006). **Table 1** also lists the main survival environment of foodborne pathogenic bacteria, including soil, freshwater, seawater, raw food, organisms, and even dust. Moreover, foodborne pathogens may enter the VBNC state and inhabit different stressful environments, including starvation, extreme temperature, chemical and UV-exposed environments. These findings allow readers to gain a better understanding of the various induction and resuscitation conditions of VBNC foodborne pathogens.

**Table 1:** The species of foodborne pathogenic bacteria with a proven VBNC state.

Genus	Name of strains	Survival environment	Induced condition	Resuscitation condition	Reference
**Gram-negative bacteria**		
*Aeromonas*	*A. hydrophila*	Aquatic environments (freshwater)	Starvation, low temperature, salt stress	Liquid media with sodium pyruvate, temperature upshift	Pianetti et al., 2012
*Brucella*	*B. melitensis*	Livestock	Metallic copper surfaces	–	Borkow, 2014
*Burkholderia*	*B. pseudomallei*	Soil, water	High temperature, low pH, osmotic stress, UV exposure	–	Inglis and Sagripanti, 2006
*Campylobacter*	*C. coli*	Intestinal tract of chicken	Starvation, low pH, low temperature	Embryonated chicken eggs	Chaveerach et al., 2003
	*C. jejuni*	Untreated water, raw milk, poultry meat	Oxygen-rich conditions, low temperature in nutrient-rich conditions	Mouse model, embryonated eggs	Chaveerach et al., 2003; Chaisowwong et al., 2012; Patrone et al., 2013; Magajna et al., 2015; Oh et al., 2015
*Citrobacter*	*C. freundii*	–	–	–	Rowan, 2004
*Edwardsiella*	*E. tarda*	Freshwater and marine fish	Starvation seawater, low temperature	Chick embryos, nutrition with temperature upshift	Du et al., 2007b
*Cronobacter*	*C. sakazakii*	Milk product	Dry Stress	–	Barron and Forsythe, 2007
*Escherichia*	*E. coli* O157:H7 EHEC	Vegetables and drinking water	Starvation, low temperature, UV exposure, high pressure carbon dioxide HPCD	Fresh Tryptic Soytone Broth, temperature upshift	Dinu and Bach, 2013; Zhao F. et al., 2013; Zhang et al., 2015
	*E. coli* O104:H4	Enugreek sprouts and seeds	Starvation copper ions or tap water	Stress relief and plated on rich agar medium	Aurass et al., 2011
	*E. coli* ST64111 ETEC	Water environments ponds, rivers, lakes and sea	Starvation seawater and freshwater	–	Lothigius et al., 2010
	*E. coli* H10407 EPEC	Marine environment	Starvation seawater	–	Pommepuy et al., 1996
*Pseudomonas*	*P. fluorescens*	Soil, water, poultry meat	Starvation, chemicals chlorination, high temperature	–	Peneau et al., 2007
	*P. putida*	–	–	–	Rowan, 2004
*Salmonella*	*S. agona*	Feed processing environment	Desiccation	–	Habimana et al., 2014
	*S. choleraesuis*	Intestines of pigs	Starvation	–	Bogosian et al., 1998; Hsueh et al., 2004
	*S. enteritidis*	Poultry and eggs	Rich medium with H_2_O_2_	M9 liquid medium with pyruvate	Braden, 2006; Morishige et al., 2013
	*S. enterica* serovar Oranienburg	Pickled foods	Starvation 7% NaCl at 37°C	Recombinant Rpf rRpf proteins	Panutdaporn et al., 2006
	*S. typhi*	Groundwater, vegetable surface	Low temperature, copper ions	Rich medium with 1% catalase or 3% Tween 20	Zeng et al., 2012
	*S. typhimurium*	Intestines of poultry, livestock and mouse	Starvation, low temperature, desiccation	Rich medium, temperature upshift	Gupte et al., 2003; Habimana et al., 2014
*Shigella*	*S. dysenteriae*	Utensils used in eating and drinking glasses	MacConkey agar with fomites, low temperature	Rich medium, temperature upshift	Rahman et al., 1996
	*S. sonnei*	Vegetable surfaces, raw ground beef	–	–	Oliver, 2005
*Vibrio*	*V. alginolyticus*	Seafood, seawater	Starvation, low temperature	Rich medium, temperature upshift	Albertini et al., 2006; Du et al., 2007a
	*V. cholera*	Estuarine and brackish water	Starvation, low temperature, oxygen	Temperature upshift, co-cultivation with HT-29 cells	Rao et al., 2014; Fernández-Delgado et al., 2015; Imamura et al., 2015
	*V. parahaemolyticus*	Estuarine water, seafood	Starvation, low temperature	Rich medium, temperature upshift	Bates and Oliver, 2004; Hung et al., 2013
	*V. vulnificus*	Raw and undercooked shellfish, seafood	Oxidative stress, low temperature, starvation	Rich medium, temperature upshift, quorum sensing QS signal molecule AI-2	Abe et al., 2006; Ayrapetyan et al., 2014; Rao et al., 2014
*Yersinia*	*Y. pseudotuberculosis*	Warm- blooded organisms domestic animals and birds	Starvation, low temperature	Rich medium	Pawlowski et al., 2011
	*Y. enterocolitica*	Natural water, soil, animal manure, refrigerated foods	–	–	Pawlowski et al., 2011
**Gram-positive bacteria**		
*Bacillus*	*B. cereus*	Soil, cereals	Pulsed electric field	–	Rowan, 2004
*Clostridium*	*C. perfringens*	Crops and vegetables	–	–	Rahman and Noor, 2012
*Enterococcus*	*E. faecalis*	Intestines of animals	Starvation, low temperature, high pH	Rich medium, temperature upshift	Lleo et al., 2001; Jiang et al., 2014
	*E. faecium*	Intestines of animals	Starvation, low temperature	Rich medium, broth with sodium pyruvate	Lleo et al., 2001
*Listeria*	*L. monocytogenes*	Raw food milk, meat, vegetables, sausages and seafood, ready-to-eat RTE food	Starvation, low pH, low temperature or not, chemicals food preservatives, pulsed electric field	NOT rich medium with/without sodium pyvurate, embryonated eggs	Rowan, 2004; Cappelier et al., 2007; Cunningham et al., 2009; Gião and Keevil, 2014; Xuan et al., 2017
*Mycobacterium*	*M. avium*	Drinking water distribution systems, household plumbing	–	–	Radomski et al., 2010
*Staphylococcus*	*S. aureus*	A wide range of environments water, dry dust and mammalian abscesses	Starvation, low temperature, antibiotic vancomycin or quinupristin/dalfopristin	Rich medium with sodium pyruvate, temperature upshift	Masmoudi et al., 2010; Pasquaroli et al., 2013, 2014; Li et al., 2016

*“–” means unknown.*

## Characteristics of VBNC Cells

Although VBNC bacteria lose culturability on normal culture medium, this does not mean that these cells are equivalent to dead cells. For example, the membrane of dead cells is damaged so that the genetic material in the cell cannot be preserved and expressed, while VBNC cells have a complete membrane structure that ensure the genetic information is not lost (Lahtinen et al., 2008). Moreover, while dead cells lose absorptive capacity and are metabolically inactive, Rahman et al. (1994) confirmed that VBNC *Shigella* can take up methionine for protein synthesis, thus demonstrating that bacteria in the VBNC state can exchange outside material.

Although VBNC bacterial cells have many common features with culturable cells, a series of physiological changes occur during the transition from the normal state to the VBNC state, including slowing down of the absorption of nutrients and reduction of the level of macromolecular synthesis and metabolism, the concentration of the cytoplasm and total proteins (Jeffreys et al., 1998). In summary, these variations encompass cellular morphology, metabolism, stress tolerance, gene expression and potential virulence, and formation mechanism of VBNC cells.

### Cellular Morphology

Regarding cellular morphology, VBNC cells maintain apparent cell integrity but exhibit dwarfing (Costa et al., 1999). Apart from cell dwarfing, researchers have also observed cell rounding in VBNC state of many species, with a reduced size and varied spherical shape (Adams et al., 2003). VBNC cells of *Salmonella typhi* exhibiting metabolic activity were decreased in size and coccoid in shape compared with the normal rod-shaped cells (Zeng et al., 2012). *Edwardsiella tarda* changes from a 1.9 μm × 1.1 μm short rod to a coccoid shape with an average radius of 0.5 μm in the VBNC state (Du et al., 2007b). *V. parahaemolyticus* also changes from rods in the exponential phase to cocci in the VBNC state, and concurrently the cell walls become looser and more flexible at the initial stage, followed by the formation of a new thin wall (Su et al., 2013). Although most bacteria entering the VBNC state are reduced in size, some Gram-positive bacteria become larger, such as *Enterococcus faecalis*, in a low temperature and low nutrient environment and are slightly elongated (Signoretto et al., 2000). Moreover, it is worth noting that the morphological change from rod to ball does not necessarily appear in all VBNC state bacteria, some VBNC cells have been confirmed to remain intact or to exhibit a spiral morphology at lower temperatures (Lázaro et al., 1999). However, these changes in morphology do not reveal whether a bacterium is in the VBNC state because they also commonly occur in non-VBNC cells (Li et al., 2014).

The VBNC cell wall and membrane differ from normal cells, which could be a manifestation of the physiological state associated with maintaining viability. An increase in the percentage of short and long chain fatty acids and a reduction in the main membrane lipid content (C16, C16:1, C18) were observed in VBNC cells of *Vibrio vulnificus* by Linder K (Linder and Oliver, 1989). These results show that changes in fatty acids play an important role in the protection of cell membrane fluidity during environmental stress. In terms of proteins, an obvious reduction in membrane protein (6.3 kDa) was observed in VBNC *Staphylococcus aureus* (Trudeau et al., 2012). Moreover, after entering the VBNC state, the structure of the peptide in the cell wall displayed relatively large changes, and Signoretto et al. (2002) also discovered that peptidoglycan DAP–DAP cross-linking in VBNC cells of *E. coli* increased more than three times. These findings indicate that the ability of VBNC cells to resist external mechanical damage is greatly improved.

### Metabolic Activity

Bacterial cells in the VBNC state maintain metabolic activity in harsh environments (Du et al., 2007a), and the energy of VBNC bacteria is mainly supplied by branched chain amino acids under starvation conditions (Ganesan et al., 2007). However, reductions in the percentage of total lipids, carbohydrates and poly-β-hydroxybutyrate were detected in starved *Vibrio cholera*, which indicated that these large molecules could be used as the primary energy source to maintain the survival of cells (Clements and Foster, 1998). A reduction in DNA was also observed in VBNC cells (Jeffreys et al., 1998). In addition, Trevors et al. (2012) found that the contents of nucleic acid molecules in the cytoplasm of starved bacteria and VBNC bacteria were lower than those in normal bacteria. Nevertheless, the mechanism underlying the decrease in DNA content in VBNC cells remains unclear and necessitates further research.

Although VBNC cells are similar to starved cells, their protein expression levels are different. Lai et al. (2009) showed an increase in protein content in VBNC cells of *V. parahaemolyticus* ST550, and the protein content in starved cells was significantly reduced. Either the upregulation of VBNC cell protein or the downregulation of starved cell protein can suppress the exponential phase of *V. parahaemolyticus* and lead to entry into the VBNC state.

### Stress Tolerance

Compared with culturable cells, VBNC cells have greater physical, chemical, and antibiotic resistance, which might be due to their lower metabolic activity and stronger cell wall strengthened resulting from the increased peptidoglycan cross-linking (Signoretto et al., 2000). To study the VBNC state, Nowakowska and Oliver (2013) developed a model organism from *V. vulnificus.* Using this model, VBNC cells of *V. vulnificus* can withstand a variety of stresses while dormant, including high doses of antibiotics, toxic heavy metals, high temperatures, high salinity, ethanol, and acid. In terms of chemical stress, a similar study conducted in *V. parahaemolyticus* showed that VBNC cells were resistant H_2_O_2_ and low salinity but remained sensitive to bile salts (Wong and Wang, 2004). In terms of antibiotic stress, the VBNC state of several foodborne pathogens such as *E. coli* O157, *S. aureus, V. vulnificus*, and *C. jejuni* have been found to be resistant to several antimicrobials (Ramamurthy et al., 2014). *S. aureus* can enter the VBNC state in infectious biofilms and the presence of vancomycin or quinupristin/dalfopristin can inadvertently induce a true VBNC state or persistence in *S. aureus* cells embedded in biofilms, suggesting a role for *staphylococcal* biofilms in recurrent infections (Pasquaroli et al., 2013). During food pasteurization and preservation processes, certain bacterial cells can be induced to enter the VBNC state (Zhao F. et al., 2013; Kramer and Muranyi, 2014). In general, VBNC cells are resistant to multiple antimicrobials, which may cause treatment failure at times (Hu and Coates, 2012). In fact, a recent study found that foods subjected to antimicrobial treatment harbored considerable numbers of VBNC cells (Anvarian et al., 2016). The presence of VBNC cells is considered a threat to human health and food safety due to the shortened shelf life that cause early spoilage of food products (Ayrapetyan and Oliver, 2016). Although VBNC cells of foodborne pathogens have been shown to resist a range of stresses, with respect to food safety, more research examined VBNC cell resistance to specific food treatments, such as special processing, preservation and packaging techniques, is warranted (Ayrapetyan and Oliver, 2016).

### Gene Expression and Potential Virulence

Bacteria in the VBNC state retain the ability to express multiple genes. Yaron and Matthews (2002) discovered many genes that could be expressed in the VBNC state of *E. coli* O157:H7, including *mob*A, *rfb*E, *stx*1, *stx*2 and some genes related to the synthesis of 16s rRNA. Recently, Patrone et al. (2013) detected the expression of the protein gene *Cad* F in VBNC cells of *C. jejuni* ATCC 33291 and *C. jejuni* 241 by RT-PCR, and observed that the *C. jejuni* VBNC population maintained an ability to adhere intestinal cells. After entering the VBNC, *Helicobacter pylori* can continue to express *Mur* G, which is a kind of sugar-based transfer enzyme that has been confirmed to be necessary for the peptidoglycan recombination that occurs in VBNC state *E. coli* (Signoretto et al., 2002). These studies demonstrated that virulence genes in VBNC cells can be expressed and the synthesis of metabolites carried out normally.

*Escherichia coli* O157: H7 is one of the most important foodborne pathogenic bacteria, arising mainly from A/E damage on the surface of intestinal epithelial cells via the production of Shiga toxin, haemolysin and adhesin, which can cause diseases such as diarrhea, haemorrhagic colitis, and haemolytic-uremic syndrome. Yaron and Matthews (2002) found that the toxin genes (*stx1* and *stx2*) could still be expressed in the VBNC state of *E. coli* O157:H7 by reverse transcription PCR. Moreover, in a study examining food safety risk factors, Dinu and Bach (2011) also discovered that VBNC *E. coli* O157: H7 exhibited the potential virulence, and stable expression of the toxin gene (*hly, stx*1, and *stx*2) and the production of enterotoxin were observed in VBNC cells. In addition, the VBNC cells of *C. jejuni* also retained the ability to invade human intestinal epithelial cells (Chaisowwong et al., 2012). However, interestingly, expression of the virulence gene in VBNC cells does not necessarily indicate that the cells will produce toxins. Lothigius et al. (2010) found that although enterotoxigenic *E. coli* (ETEC) entering the VBNC state maintain the expression of virulence genes *eltB* and *estA* encoding the LT and STh enterotoxins, enterotoxins were not produced as determined using GM1-ELISA methods. Nonetheless, there is the potential danger in VBNC cells that are still pathogenic and even cause fatal diseases, which may be due to rapid resuscitation in suitable condition (Du et al., 2007b).

### Mechanism of VBNC Cell Formation

Since the concept of the VBNC was proposed, a great deal of literature has been published on the VBNC state, although most of them have concentrated on biological characteristics. Thus, the mechanism by which the VBNC state occurs in bacteria is still not well understood (Pinto et al., 2015). The present formation mechanism of the VBNC state has been hypothesized as follows. First, the extreme conditions can lead to poor-quality cells, resulting in a loss of cell activity so that the VBNC cells cannot be cultured (Nyström, 2003). Second, the VBNC state exhibits a tendency not to die but exhibits a survival strategy, with procedural responses to adapt to adverse environments (Oliver, 2010). Third, this hypothesis is currently supported by the finding that non-culturable VBNC cells are the result of gene regulation (Ayrapetyan et al., 2015).

Although the molecular mechanism underlying the formation of the VBNC state is not fully understood, several genes that exhibit importance in the VBNC state have also been found to play integral roles in the formation of VBNC cells. Here, it is worth discussing the *rpoS* gene. The major stress regulator, RpoS (σ^S^), which is expressed by the *rpoS* gene, is a stationary-phase sigma factor that allows bacteria to survive under different environmental stresses (such as acidic conditions, high osmotic pressure, oxidation, and starvation) (Bhagwat et al., 2006). This capability implies that RpoS can improve the ability of cells to adapt to the environment and thus hinder formation of the VBNC state. It has been shown that *rpoS* mutants lacking ppGpp more rapidly enter the VBNC state than normal strains (Boaretti et al., 2003). Additionally, Kusumoto et al. (2012) showed a reduced RpoS level during VBNC induction of *Salmonella*, and RpoS indeed delayed the formation of VBNC cells. Although RpoS is an obstacle for the formation of the VBNC state, it has been reported that VBNC cells continue to express the *rpoS* gene (Smith and Oliver, 2006). It is possible that VBNC cells must regulate RpoS, which is important for the maintenance of resistance and persistence under stresses. Boaretti et al. (2003) confirmed this hypothesis and found that the *rpoS* mutants lost culturability and died earlier than VBNC cells of their *E. coli* parental strains, suggesting that the *rpoS* gene is closely related to persistence in the VBNC state. These findings indicate that although the expression and regulation of the *rpoS* gene significantly hinder the formation of VBNC cells, long-term survival of VBNC cells is not possible in the absence of RpoS protein. However, a contrasting viewpoint has been proposed. This opposing perspective is that (p)ppGpp modulated by protein RelA may be an inducer of the VBNC state and that cells lacking ppGpp are less likely to enter the VBNC state (Ayrapetyan et al., 2015). It is known that (p)ppGpp is a regulatory signaling molecule that can regulate RpoS, and the elevated level of (p)ppGpp will lead to a several-fold increase in the amount of RpoS, which plays a crucial role in the accumulation of RpoS (Magnusson et al., 2005). Thus, the possibility that (p)ppGpp may be an inducer of the VBNC state suggests that RpoS may be an inducer that can facilitate the more rapid entry of cells into the VBNC state. Although these two views are conflicting, they both illustrate that RpoS significantly affects the formation of VBNC cells and also enhances stress resistance in VBNC cells.

**FIGURE 1:**
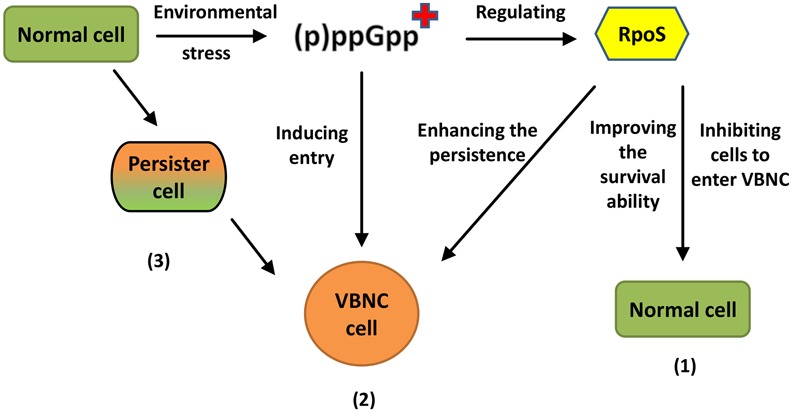
**Different views on the formation mechanism of VBNC cells.** (1) RpoS proteins significantly hinder the formation of VBNC cells but enhance the persistence of VBNC. “+” means an accumulation of (p)ppGpp. (2) (p)ppGpp may be an inducer which can make cells more quickly into VBNC. (3) The normal cell will enter the persister state before entering VBNC state.

The persister state was first described by Bigger (1944) as a multidrug-tolerant state, representing another dormancy state related to the VBNC state. In this state, the cells are not growing but can quickly regain culturability on medium. Ayrapetyan et al. (2015) argued that these two closely related states are part of a shared ‘dormancy continuum,’ suggesting that logarithmic-phase cells would enter the persister state before entering the VBNC state. Orman and Brynildsen (2013) also found that the presence of a small number of persisters could cause an accumulation of VBNC cells, and believed that the persister state is a transitory state leading to the VBNC state. The above findings offer some hypotheses regarding the VBNC formation mechanism (**Figure 1**).

The above research about the formation mechanism of VBNC mainly focused on hypotheses and reasoning. At the molecular level, the research was mainly focused on the gene or protein level with some functions, which only partly reflected the formation mechanism of VBNC state. However, the study focused on the single and individual mechanisms, the lack of a comprehensive and systematic analysis. Most of the literatures to a single transcriptomics or proteomics approaches, the lack of integration analysis of the both, it is difficult to achieve information complement and functional verification (Capozzi et al., 2016; Feng et al., 2016). With the development and application of high-throughput sequencing technology and bioinformatics, in order to comprehensively and thoroughly understand the formation mechanism of VBNC state, researchers will use omics technology to study the formation of VBNC state mechanism.

## Detection of VBNC Cells

At present, the method used to detect VBNC cells is mainly based on two key characteristics of VBNC cells: viability and non-culturability. Generally, if bacteria lose culturability but are still viable, they can be considered to have entered the VBNC state. Thus, using the conventional plate counting technique to confirm the cells in a non-culturable state is the first major step used to detect VBNC cells, followed by the estimation of viable cells using other methods.

One common method is based on microscopic enumeration with staining procedure to directly detect the viable cells, including the direct counting method of viable bacterial cells (DVC) based on the substrate absorption ability (Kogure et al., 1979), the respiration detection method (such as CTC or INT) based on the ability of the electron transport system (Albertini et al., 2006) and the LIVE/DEAD^®^ BacLight^TM^ fluorescence staining method based on the cell membrane structure integrity (Cunningham et al., 2009).

The second method is based on molecular diagnostic approaches to detect gene expression or gene amplification selectively, such as reverse transcription PCR, quantitative real-time polymerase chain reaction (qRT-PCR) and loop-mediated isothermal amplification (LAMP). Bacterial mRNAs have been proposed as markers for cell viability because they are very unstable molecules with very short half-lives inside the cell (Sheridan et al., 1998). Thus, it would be expected that as long as VBNC bacteria are alive, they should produce some mRNA molecules. Reverse transcription PCR is commonly used in many bacterial species to determine the viability of cells (Trevors, 2011). Selective gene amplification is an emerging approach to detect viable cells. Using this approach, viability is based on membrane integrity. Propidium monoazide (PMA) or ethidium monoazide (EMA) is a high-affinity photolysis DNA nucleic acid dye that can only enter cells with damaged membranes (considered ‘dead’) and bind covalently to cellular DNA through visible-light photocatalysis, whereas the intact membranes of ‘live’ cells pose a barrier to this molecule. The PMA treatment is followed by extraction of genomic DNA and its analysis by quantitative PCR or LAMP. The covalent cross-linkage of PMA to DNA has been shown to result in a strong inhibition of PCR amplification of the modified DNA. The result of treatment is that only unmodified DNA from intact cells containing DNA that is not cross-linked to PMA can be amplified, whereas PCR amplification of modified DNA from membrane-compromised cells is efficiently suppressed (Nocker and Camper, 2009). Dinu and Bach (2013) accurately detected *E. coli* O157:H7 VBNC cells on the surface of lettuce and spinach plants by PMA-qPCR, which provided a detection limit of 10^3^ CFU/g leaf, suggesting that PMA-qPCR was an appropriate technique to detect the VBNC cells of foodborne pathogens in contaminated vegetables. PMA can also be combined with LAMP. Zhao X. et al. (2013) developed a PMA-LAMP assay and selectively detected viable *E. coli* O157 cells within 1 h by PMA-LAMP. Zhong et al. (2016) also developed a real-time fluorescence LAMP technique combined with PMA, and applied it for the quantitative detection of VBNC *V. parahaemolyticus*. Lin et al. (2016) also adopted qRT-PCR and PMA-qPCR to observe the potential induction of VBNC cells by water reclamation processes.

The other methods focus on the identification of VBNC cells using biological sensors, especially gene sensors and receptor sensors that are biosensors based on DNA (Paniel et al., 2013). A DNA or RNA target gene sensor method is applied mainly to detect the hybridization reaction between DNA or RNA sensors and single-stranded DNA during sample identification. Regarding the receptor sensor method, DNA or RNA can be used as a receptor to achieve high affinity and specificity in combination with the target molecules. It is worth noting that one should first select the appropriate biological recognition elements and sensing format when adopting this method to achieve the desired objectives. However, less research has been conducted in this area, and further validation is needed.

Recently, novel detection methods have also developed for separating cells based on their physiological states (Ayrapetyan and Oliver, 2016). Fluorescence techniques combined with direct optical detection methods for the rapid assessment of bacterial viability have been increasingly followed for several years. Among these techniques, flow cytometry (FCM) has been shown to be a powerful tool for rapidly analyzing populations on a cell-by-cell basis and can be applied in many areas of food safety or medical microbiology (Léonard et al., 2016). The main principle is that particles in suspension are pumped into a narrow flow stream intersected by one or more laser beams. Single particles, such as microbial cells, are illuminated individually with the resulting light scatter and fluorescence emission detected at appropriate wavelengths (Bridier et al., 2015). Bridier et al. (2015) summarized the applications of FCM in food microbiology such as study of food bacteria function, detection of food microbial communities or detection and persistence of foodborne pathogens. Recently, Mathur et al. (2016) reviewed the advancement of FCM and the introduction of novel fluorochromes allow to study the viability of cells, the membrane structure and its integrity, and the membrane potential at a single-cell level. The ability to use FCM to visualize, enumerate and analyze a population of cells into subpopulations of varying physiological status is a valuable aid to understanding this intricate area for the microbiologists (Léonard et al., 2016). Indeed, the use of personalized probes and dyes for the detection of changes in specific targets and intracellular activities permits the targeted use of FCM to ascertain the structural and functional characteristics of a population of VBNC cells.

## Induction of VBNC Cells

During the process of food processing and storage, there are numerous factors that induce foodborne pathogens to enter into the VBNC state. In general, the factors (physical and chemical) create worse bacteria growth conditions that may stress the bacterial cells into the VBNC state. The physical factors that induce the VBNC state of bacteria mainly include low/high temperature (Dinu and Bach, 2013), drying (Barron and Forsythe, 2007), irradiation (Zhang et al., 2015), oxidative stress (Oh et al., 2015), starvation (Lothigius et al., 2010), a pulsed electric field (Rowan, 2004), pulsed light and high pressure carbon dioxide (HPCD) (Feng et al., 2016). The chemical factors include food preservatives and disinfectants (Oliver, 2010; Ding et al., 2016).

To explore the physical induction factors, many researchers have carried out relevant simulation experiments (Rowan, 2004; Barron and Forsythe, 2007; Dinu and Bach, 2013; Patrone et al., 2013; Zhao F. et al., 2013). For example, the major foodborne pathogens, such as *E. coli* O157, *C. jejuni, V. parahaemolyticus, L. monocytogenes*, and *S. aureus*, have been validated to enter the VBNC state under low temperatures conditions (Bates and Oliver, 2004; Masmoudi et al., 2010; Chaisowwong et al., 2012; Dinu and Bach, 2013; Patrone et al., 2013; Gião and Keevil, 2014; Liu et al., 2016). In addition to low temperature factors, cells entering the VBNC state have been detected during high temperature sterilization processes such as the pasteurization of milk (Gunasekera et al., 2002). Regarding other physical factors, the VBNC cells of *Cronobacter sakazakii* have been detected in milk products as a result of dry stress (Barron and Forsythe, 2007). *C. jejuni* (Oh et al., 2015) and *V. vulnificus* (Abe et al., 2006) have been confirmed to enter the VBNC state under oxidative stress. *L. monocytogenes* and *B. cereus* entered the VBNC state by treatment with a pulsed electric field (Rowan, 2004). The cells of *E. coli* O157:H7 were induced into a VBNC state by UV disinfection (Zhang et al., 2015). A large number of *E. coli* were also observed to enter the VBNC state after pulsed light treatment, and some of them exhibited metabolic loss and cell membrane damage (Kramer and Muranyi, 2014). These findings all support the idea that various kinds of physical stress factors during food processing may induce foodborne pathogens to enter the VBNC state.

Some chemical reagents are commonly used in the process of food processing, such as the addition of food preservatives to extend the shelf life of food or of disinfectants in the processing plant and equipment for disinfection. In the food industry, pathogens with a certain degree of resistance are created by the indiscriminate use of disinfection solution (Meyer, 2006). Some pathogens leave behind and build up a resident flora on surfaces after cleaning and disinfection, and partial cells from the gradual accumulation of resident flora can be induced to enter a VBNC state (Peneau et al., 2007). Such chemical reagents were once used indiscriminately, not only cannot improve food safety but also to induce bacteria to enter the VBNC state. Researchers have shown that the indiscriminate use of preservatives may be a threat to public health. For example, although potassium sorbate is a kind of commonly used broad-spectrum antimicrobial agent, Cunningham et al. (2009) found that the cells of *L. monocytogenes* grown in the presence of potassium sorbate at pH 4.0 entered a VBNC state within 24 h. They also indicated that temperature had a significant impact on the ability of potassium sorbate to induce VBNC cells, which were observed at 37°C but not at 4°C or 21°C. However, the wide use of chlorinating disinfectants in food processing workshops may also cause bacteria to enter the VBNC state. Peneau et al. (2007) simulated meat processing plants in the laboratory and adopted the same disinfection methods used in the factory to disinfect production equipment. They found VBNC cells of *Pseudomonas fluorescens* on the production equipment. It has also been reported that excessive use of disinfectants induce pathogenic bacteria, such as *E. coli* and *C. jejuni*, to enter the VBNC state (Meyer, 2006). Moreover, because of the use of chlorinating disinfectants, bacteria that are present in tap water may also enter the VBNC state. Al-Qadiri et al. (2011) confirmed the presence of VBNC state *E. coli* O157:H7 and *C. jejuni* in tap water by molecular biological detection. Therefore, it is important to pay attention to the rational use of disinfectants during the process of food safety production.

## Resuscitation of VBNC Cells

### What Is Resuscitation?

The term “resuscitation” was first presented by Roszak et al. (1984) to describe the recovery of VBNC cells of *Salmonella enteritidis*. It is an important feature of VBNC cells, and the recovered cells display improved metabolic activity and restored culturability. In fact, resuscitation is a complicated process and may not be performed by only direct removal of inducing factors. In addition, not all VBNC strains can be recovered (Rowan, 2004). The resuscitation conditions differ for different bacteria, and thus only under suitable conditions can VBNC cells achieve resuscitation. **Table 1** presents the various resuscitation conditions that have been reported for different foodborne pathogens, such as *C. jejuni*, which could be resuscitated by incubation in embryonated chicken eggs but not in rich medium. In addition, the same resuscitation method had different effects on different bacteria and even on different strains of the same species. For example, Pinto et al. (2011) discovered amino acids could resuscitate VBNC cells of haemolytic *E. coli* but not *E. coli* O157:H7.

The biggest challenge associated with the resuscitation of VBNC cells has existed for a long time: whether the culturable state of bacteria is caused by a real recovery of VBNC cells or the regrowth of residual culturable cells that are not undetected by the plate counting method (Whitesides and Oliver, 1997). The difference between residual undetected culturable cells and VBNC cells is that the former retains culturability. To date, there is no effective method to distinguish culturable cells from resuscitation cells or normal cells, and thus different views are apparent regarding the resuscitation of VBNC cells. In the resuscitation experiment of VBNC *V. cholerae*, Ravel et al. (1995) found that the number of resuscitated cells was similar to the samples diluted 10 times and 100 times, which was also approximately 2.2 × 10^5^ cfu/mL, rather than the expected decrease in presentation. Consequently, they believed that the increased number of *V. cholerae* was caused by the regeneration of residual bacterial cells but not the resuscitation of VBNC cells. Nevertheless, Whitesides and Oliver (1997) demonstrated the resuscitation of VBNC cells after 2 years by further reducing the proportion of culturable cells by serial dilutions. Thus, the resuscitation has been widely recognized.

To date, the virulence of VBNC pathogens has been proven to be recovered or maintained after resuscitation. Resuscitated VBNC cells such as *L. monocytogenes* (Cappelier et al., 2007) and *S. typhi* (Zeng et al., 2012) retain their virulence and cause varying degrees of damage to mice, leading even to death. Moreover, the resuscitated VBNC pathogens may be involved in several foodborne outbreaks, such as *E. coli* O157:H7 (Makino et al., 2000), *E. coli* O104:H4 (Aurass et al., 2011), and *Salmonella* (Asakura et al., 2002). Although there is no clear evidence to prove that resuscitated foodborne pathogens can directly cause human diseases, their security risks to public health cannot be ignored. Therefore, it is necessary to understand the factors that can promote resuscitation and thus taking effective measures to prevent the occurrence of food hazards.

### Factors that Stimulate Resuscitation

The resuscitation of VBNC cells can be triggered by a variety of stimuli factors, such as an increase in the nutrient concentration, increases or decreases in temperature, the presence of chemical stimuli and even co-cultivation with host cells. In 1984, rich medium was first used to resuscitate VBNC cells in *S. enteritidis* by Roszak et al. (1984). Since then, to identify the factors that stimulate the recovery of VBNC cells, a number of researchers have successively performed resuscitation experiments under different conditions.

An increase in temperature is a common physical stimulus to resuscitate most VBNC cells induced by low temperature, such as *E. coli* O157:H7, *A. hydrophila, S. typhimurium, S. dysenteriae, Vibrio* spp., *E. faecalis*, and *S. aureus* (**Table 1**). Resuscitation can also be mediated by different kinds of chemical stimuli, including sodium pyruvate (Lleo et al., 2001; Pinto et al., 2011; Morishige et al., 2013; Pasquaroli et al., 2013), amino acids (Pinto et al., 2011), and Tween 80 (Trinh et al., 2015). It is worth mentioning that researchers have different views on the resuscitation role of pyruvate. On the one hand, it is deliberated whether pyruvate cannot recover VBNC cells. Li et al. (2014) reported that the VBNC cells of *S. typhimurium* could not be recovered by supplementation with antioxidants such as pyruvate, catalase or oxyrase, but could be resuscitated by an autoinducer. On the other hand, some people insist that pyruvate has a significant effect on the resuscitation of VBNC strains. For example, Pinto et al. (2011) found that starving cells of *E. coli* could easily enter the VBNC state after the addition of pyruvate. Morishige et al. (2013) also discovered that the VBNC cells of *S. enteritidis* caused by H_2_O_2_ stress could regain culturability by the addition of sodium pyruvate but not pyruvate analogs (like phenyl pyruvate or bromoacetone), thus confirming that pyruvate was one of the key molecules in the process of resuscitation by triggering the synthesis of macromolecules such as DNA and protein.

In addition, VBNC cells can also be resuscitated by biological stimuli such as eukaryotic cells. Senoh et al. (2010) found that the VBNC cells of *V. cholerae* could be converted into a culturable state after co-cultivating with eukaryotic cells. However, another study showed that VBNC *C. jejuni* cells could form colonies on agar plates after co-cultivation with Caco-2 cells, but most VBNC cells could not be resuscitated (Chaisowwong et al., 2012), which indicated that the presence of host cells was a biological stimulus factor that can trigger the resuscitation of a fraction of VBNC cells. Additionally, Imamura et al. (2015) discovered a phenomenon in which VBNC cells of *V. cholerae* were initially converted into a culturable state by treatment with HT-29 cell extract or catalase but subsequently entered a state from which they could not be resuscitated. These non-resuscitated cells were verified to be viable by fluorescence microscopy and could be resuscitated by co-cultivation with HT-29 human colon adenocarcinoma cells. However, all cells entered a state from which they could not be resuscitated, even by co-cultivation with HT-29. Thus, the VBNC cells that were resuscitated by biological factors could not be maintained for a long time because the requirements for resuscitated VBNC cells changed over time such that the HT-29 cells could not always maintain resuscitation of the VBNC cells.

### Resuscitation Mechanism of VBNC Cells

**FIGURE 2:**
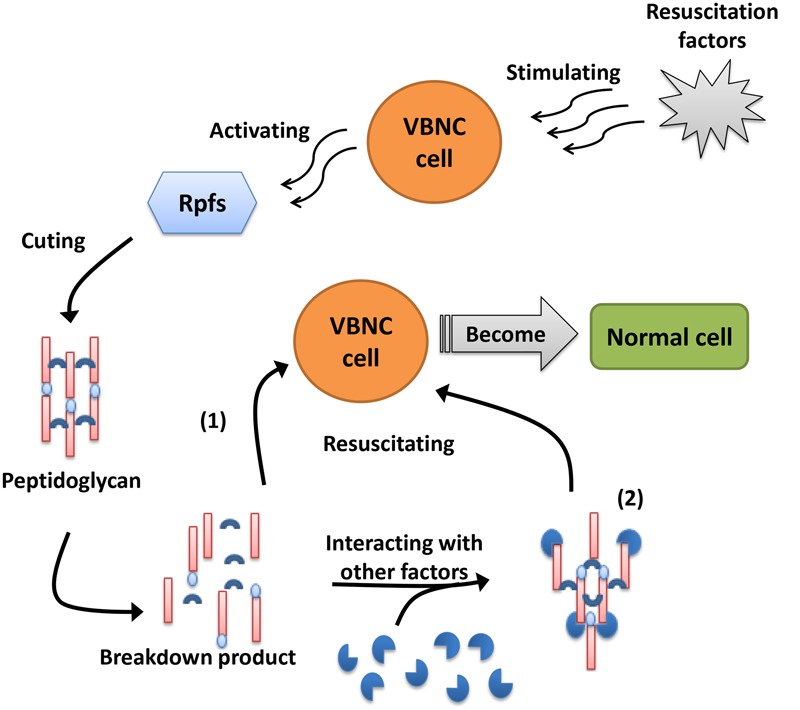
**Two viewpoints about the mechanism of Rpfs.** (1) Rpfs are required to cleave the peptidoglycans with inhibitory properties distributed in specific area of dormant cell wall and thus promote cell division and growth again. (2) The breakdown product(s) of peptidoglycan divided by Rpfs may interact with other factors and function as “second messengers” to stimulate the resuscitation and growth of VBNC cells.

Although the resuscitation mechanism of VBNC cells remains largely unknown, studies examining resuscitation from the VBNC state have provided many promising results, and people have gradually acquired a greater understanding of the resuscitation mechanism with the development of molecular biology. Resuscitation promoting factor (Rpf), a highly conserved protein composed of 220 amino acids that is directly related to the resuscitation of VBNC cells, has been demonstrated to restore the growth and reproductive ability of VBNC cells (Mukamolova et al., 1998). Rpf proteins have been shown to act as cytokines that, when secreted into the medium by growing cells, bind to the surface receptors of dormant cells and trigger resuscitation (Pinto et al., 2015). Panutdaporn et al. (2006) also showed that the growth of *S. enterica* serovar Oranienburg cells could be enhanced by a certain concentration of rRpf protein. Moreover, Pinto et al. (2011) observed that Rpf supernatant fluid treated with proteinase K could resuscitate the VBNC cells of *E. coli*, suggesting that the breakdown products of Rpf could also restore VBNC cells. In addition, Pinto et al. (2013) validated the hypothesis that two Rpfs of *L. monocytogenes*, Lmo0186 and Lmo2522, could promote resuscitation via a mechanism analogous to actinobacteria Rpf proteins. Although the mechanism of Rpf in VBNC cell resuscitation is still not well understood, most researchers believe that the mechanism of Rpf is similar to that of lysozyme, both of which play a role in hydrolysis to divide the peptidoglycan in the cell wall (Keep et al., 2006a). There are two viewpoints regarding the mechanism of Rpf (**Figure 2**): one is that the breakdown product(s) of peptidoglycan by Rpf may interact with other factors and function as ‘second messengers’ to stimulate the resuscitation and growth of VBNC cells; the other is that Rpf is required to cleave peptidoglycans with inhibitory properties that are distributed in specific areas of the dormant cell wall and thus promote cell division and growth resumption (Keep et al., 2006b). To provide insights into the regulatory mechanism of Rpf protein, Aydin et al. (2011) obtained high-level expression of recombinant *V. parahaemolyticus* YeaZ in *E. coli* to determine the atomic structure and elucidate the three-dimensional structural conservation in YeaZ homologs, which may broaden perspectives regarding the mechanism of Rpf. However, the mechanism of Rpf and its breakdown products are still not clear and require further study.

In contrast, Moorhead and Griffiths (2011) found that *C. jejuni* could respond to quorum-sensing (QS) signaling molecules (such as C4-HSL, 3OH-C4-HSL, C12-HSL, and HSL), which indicated that biofilm formation was blocked and the entry of cells into the VBNC state was delayed. It was speculated that QS signaling molecules were related to formation of the VBNC state. This conjecture was further confirmed by Ayrapetyan et al. (2014), who only found that the QS signaling molecule autoinducer-2 (AI-2) could directly awaken the VBNC cells of *V. vulnificus* but also that the AI-2 deletion mutant lost resuscitation ability from the VBNC state. Bari et al. (2013) also demonstrated that the resuscitation of dormant *V. cholera* was dramatically improved by the addition to the enrichment medium of biologically synthesized AIs, suggesting that these molecules might signal to dormant cells and then improve conditions for better growth. Furthermore, *rpoS* deletion mutant strains could not be resuscitated even with the addition of exogenous AI-2. This result shows that RpoS is not only a significant protein for VBNC formation but also an important participant in the resuscitation process mediated by AI-2.

## Public Health and Food Safety of VBNC Foodborne Pathogens

It is worth discussing whether VBNC pathogens maintain their pathogenicity if they are unable to be resuscitated. Although there is no relevant information to confirm that pathogenic bacteria that remain in the VBNC state can cause human disease, it has been reported that some VBNC pathogens retain pathogenic effects. For example, Oliver and Bockian (1995) described mice that were in a lethal state after inoculation with VBNC cells of *Vibrio vulnificus*. Amel et al. (2008) observed a fluid accumulation in the rabbit ileal loop assay (RICA) in response to VBNC *V. cholerae* O1. VBNC *Legionella pneumophila* that retained the capacity to infect the amoeba, which is its natural host (Al-Bana et al., 2014). Furthermore, one study demonstrated that VBNC *enteropathogenic E. coli* showed pathogenicity due to the continual production of enterotoxin (Pommepuy et al., 1996), and another study showed that *uropathogenic E. coli* that remained in the VBNC state may be the major causative agent of recurrent urinary tract infections in many individuals (Anderson et al., 2004). However, some VBNC pathogens have been confirmed to be non-pathogenic. Compared with culturable cells, the VBNC cells of *L. monocytogenes* were avirulent because of a failure to colonize the spleen of mice or adhere to HT-29 cells (Cappelier et al., 2007), VBNC *C. jejuni* cells were unable to colonize the caecum of newly hatched leghorn chicks (Ziprin et al., 2003) and VBNC *Salmonella typhimurium* could not infect all mice in the experiment (Habimana et al., 2014). In a word, when VBNC cells are unable to resuscitate in animals, some of them are still pathogenic and others are avirulent. The pathogenicity of VBNC cells in animals may differ due to different strains or the type of animals. To better understand the pathogenicity of VBNC cells, we propose to expand the research to include assessments of the pathogenicity of different VBNC cells and to attempt to distinguish the VBNC cells that can directly lead to disease.

Interestingly, Amel and Amina (2008) found that VBNC *S. typhimurium* cells were only recovered into culturable cells by oral administration but not by intraperitoneal injection in mice, indicating that the intestinal environment might be an essential condition for resuscitation. However, Habimana et al. (2014) demonstrated that *S. typhimurium* failed to resuscitate during passage through the gastrointestinal tract. We speculate that there may be several reasons for the different pathogenic characteristics of the same strain in mice. First, the strains of *S. typhimurium* were not the same in the two experiments; the former was LT4 and the latter was ATCC 14028. Second, the differences between the experimental animals in the two experiments may have affected the results of the experiment, such as the different genders and ages. Third, the pressure that caused the bacteria to enter the VBNC state and the induction method may determine the resuscitation ability of VBNC cells. As shown in **Table 1**, most of the VBNC cell resuscitation methods are based on the method of induction, mainly stress relief, such as *E. coli* O157:H7 induced by low temperature and the corresponding increase in temperature for resuscitation. A similar hypothesis was also proposed by Habimana, who proposed that the phenomenon was dependent on how the pathogens were originally induced into a non-culturable state (Habimana et al., 2014). The specific cause of this phenomenon is still uncertain, requiring further experiments to reach a definitive conclusion.

In the previous section (Introduction), we have described the role of VBNC pathogens in public health and food safety, and even their involvement in many foodborne outbreaks; however, there is no evidence to show that VBNC pathogens directly caused the outbreak. We propose that one of the most possible reasons is the undetectability of VBNC cells. Recent findings showed that 20% of illnesses can be linked to known pathogens, but the remaining 80% are due to unspecified or unidentified agents (Nicolò and Guglielmino, 2012), indicating that VBNC pathogens may be ignored during most outbreaks due to undetectability. For example, in 2011 in Germany, there was a large outbreak caused by an *E. coli* O104:H4 strain expressing genes characteristic of enterohemorrhagic (EHEC) and enteroaggregative *E. coli* (EAEC), involving more than 3000 cases of bloody diarrhea and haemolytic uremic syndrome. Unfortunately, the local detection department failed to detect or isolate the *E. coli* O104:H4 strain from the source of contamination until a small amount of the pathogens were isolated from the patients (Aurass et al., 2011; Scheutz et al., 2011). We suggest that the main reason for the failure to isolate the *E. coli* O104:H4 outbreak strain is that this outbreak strain entered a non-culturable state. Aurass et al. (2011) also confirmed that the *E. coli* O104:H4 outbreak strain induced by copper ions or tap water of outbreak area entered the VBNC state and was resuscitated to become a potentially pathogenic bacterium by stress relief. This finding implies that VBNC *E. coli* O104:H4 may invade the human body through contaminated food, undergo resuscitation and thus lead to disease.

The presence of VBNC cells in food is widely documented (Rowan et al., 2015). Food is frequently exposed to a complex environmental system, in which physiochemical characteristics (pH, *a*_w_, disinfectant and chemical composition) and environmental factors (high pressure CO_2_, elevated temperatures, storage temperature and time, decontamination treatments, pasteurization and packaging under modified atmosphere) act simultaneously on contaminating bacteria leading to the VBNC state. This alone poses a significant risk to the public health and food safety, as these bacteria cannot be detected by commonly used techniques (Fakruddin et al., 2013). This risk is made even greater by the fact that VBNC cells can resuscitate within the human host (Ayrapetyan and Oliver, 2016). Furthermore, studies have proven that VBNC cells of foodborne pathogens, continue to produce virulence factors in food (Dinu and Bach, 2011). These studies indicate that more effective methods for detection of foodborne pathogen must be employed, to tackle the threat posed by VBNC bacteria with regard to public health and food safety.

## Conclusion

After decades of research, the VBNC concept has attracted great attention for a variety of foodborne pathogens and the corresponding adaptation mechanisms. It seems clear that the conditions, factors and regulators during the induction and resuscitation of the VBNC state play prominent roles in some strains. However, the formation and resuscitation mechanism of the VBNC state remain unclear and thus require further study. The abilities of VBNC cells to evade detection by conventional plate counting techniques, to tolerate stressful environments including food pasteurization processes and antibiotics, and to resuscitate with virulence and cause disease could pose a great threat to food safety and infectious disease prevention. Therefore, the development of rapid, sensitive, cost-effective, and easy-to-operate methods for detection of the VBNC state is an urgent need. In conclusion, the potential application of fundamental research examining the VBNC state is very important to prevent foodborne infections, protect food safety and identify new treatments to reduce the risk of disease caused by foodborne pathogens.

## Author Contributions

XZ, JZ, and TD wrote the manuscript. CW and C-WL participated in its organization and helped to draft the manuscript. XZ, JZ, and TD revised the manuscript critically for important intellectual content. All authors read and approved the final manuscript.

## Conflict of Interest Statement

The authors declare that the research was conducted in the absence of any commercial or financial relationships that could be construed as a potential conflict of interest.

## References

[B1] Abe A. Ohashi E. Ren H. Hayashi T. Endo H. (2006). Isolation of a viable but non-culturable suppression mutant of *Vibrio vulnificus*: role of antioxidant enzymes in surviving stationary phase and low temperatures. *Fish. Sci.* 72 656–664. 10.1111/j.1444-2906.2006.01196.x

[B2] Adams B. L. Bates T. C. Oliver J. D. (2003). Survival of *Helicobacter pylori* in a natural freshwater environment. *Appl. Environ. Microbiol.* 69 7462–7466. 10.1128/AEM.69.12.7462-7466.2003 14660399 PMC310012

[B3] Al-Bana B. H. Haddad M. T. Garduño R. A. (2014). Stationary phase and mature infectious forms of *Legionella pneumophila* produce distinct viable but non-culturable cells. *Environ. Microbiol.* 16 382–395. 10.1111/1462-2920.12219 23968544

[B4] Albertini M. C. Accorsi A. Teodori L. Pierfelici L. Uguccioni F. Rocchi M. B. (2006). Use of multiparameter analysis for *Vibrio alginolyticus* viable but nonculturable state determination. *Cytom. Part A* 69 260–265. 10.1002/cyto.a.20263 16528721

[B5] Al-Qadiri H. M. Lu X. Al-Alami N. I. Rasco B. A. (2011). Survival of *Escherichia coli* O157: H7 and *Campylobacter jejuni* in bottled purified drinking water under different storage conditions. *J. Food. Protect.* 74 254–260. 10.4315/0362-028X.JFP-10-368 21333145

[B6] Amel B. K.-N. Amine B. Amina B. (2008). Survival of *Vibrio fluvialis* in seawater under starvation conditions. *Microbiol. Res.* 163 323–328. 10.1016/j.micres.2006.06.006 16870413

[B7] Amel D. Amina B. (2008). Resuscitation of seventeen-year stressed *Salmonella typhimurium*. *Oceanol. Hydrobiol. Stud.* 37 69–82. 10.2478/v10009-007-0038-x

[B8] Anderson M. Bollinger D. Hagler A. Hartwell H. Rivers B. Ward K. (2004). Viable but nonculturable bacteria are present in mouse and human urine specimens. *J. Clin. Microbiol.* 42 753–758. 10.1128/JCM.42.2.753-758.2004 14766848 PMC344478

[B9] Anvarian A. H. Smith M. P. Overton T. W. (2016). The effects of orange juice clarification on the physiology of *Escherichia coli*; growth-based and flow cytometric analysis. *Int. J. Food. Microbiol.* 219 38–43. 10.1016/j.ijfoodmicro.2015.11.016 26705746

[B10] Asakura H. Makino S.-I. Takagi T. Kuri A. Kurazono T. Watarai M. (2002). Passage in mice causes a change in the ability of *Salmonella enterica* serovar Oranienburg to survive NaCl osmotic stress: resuscitation from the viable but non-culturable state. *FEMS. Microbiol. Lett.* 212 87–93. 10.1111/j.1574-6968.2002.tb11249.x 12076792

[B11] Aurass P. Prager R. Flieger A. (2011). EHEC/EAEC O104: H4 strain linked with the 2011 German outbreak of haemolytic uremic syndrome enters into the viable but non-culturable state in response to various stresses and resuscitates upon stress relief. *Environ. Microbiol.* 13 3139–3148. 10.1111/j.1462-2920.2011.02604.x 21951606

[B12] Aydin I. Dimitropoulos A. Chen S. H. Thomas C. Roujeinikova A. (2011). Purification, crystallization and preliminary X-ray crystallographic analysis of the putative resuscitation-promoting factor YeaZ. *Acta. Crystallogr. F* 67 604–607. 10.1107/S1744309111010219 PMC3087651 21543872

[B13] Ayrapetyan M. Oliver J. D. (2016). The viable but non-culturable state and its relevance in food safety. *Curr. Opin. Food Sci.* 8 127–133. 10.1016/j.cofs.2016.04.010

[B14] Ayrapetyan M. Williams T. C. Oliver J. D. (2014). Interspecific quorum sensing mediates the resuscitation of viable but nonculturable vibrios. *Appl. Environ. Microbiol.* 80 2478–2483. 10.1128/AEM.00080-14 24509922 PMC3993182

[B15] Ayrapetyan M. Williams T. C. Oliver J. D. (2015). Bridging the gap between viable but non-culturable and antibiotic persistent bacteria. *Trends Microbiol.* 23 7–13. 10.1016/j.tim.2014.09.004 25449050

[B16] Bari S. M. Roky M. K. Mohiuddin M. Kamruzzaman M. Mekalanos J. J. Faruque S. M. (2013). Quorum-sensing autoinducers resuscitate dormant *Vibrio cholerae* in environmental water samples. *Proc. Natl. Acad. Sci. U.S.A.* 110 9926–9931. 10.1073/pnas.1307697110 23716683 PMC3683778

[B17] Barron J. C. Forsythe S. J. (2007). Dry stress and survival time of *Enterobacter sakazakii* and other *Enterobacteriaceae* in dehydrated powdered infant formula. *J. Food Prot.* 70 2111–2117. 10.4315/0362-028X-70.9.2111 17900090

[B18] Bates T. C. Oliver J. D. (2004). The viable but nonculturable state of Kanagawa positive and negative strains of *Vibrio parahaemolyticus*. *J. Microbiol.* 42 74–79. 15357298

[B19] Bhagwat A. A. Tan J. Sharma M. Kothary M. Low S. Tall B. D. (2006). Functional heterogeneity of RpoS in stress tolerance of enterohemorrhagic *Escherichia coli* strains. *Appl. Environ. Microbiol.* 72 4978–4986. 10.1128/AEM.02842-05 16820496 PMC1489321

[B20] Bigger J. (1944). Treatment of staphylococcal infections with penicillin by intermittent sterilisation. *Lancet* 244 497–500. 10.1016/S0140-6736(00)74210-3

[B21] Boaretti M. Del Mar Lleò M. Bonato B. Signoretto C. Canepari P. (2003). Involvement of rpoS in the survival of *Escherichia coli* in the viable but non-culturable state. *Environ. Microbiol.* 5 986–996. 10.1046/j.1462-2920.2003.00497.x 14510852

[B22] Bogosian G. Morris P. J. O’Neil J. P. (1998). A mixed culture recovery method indicates that enteric bacteria do not enter the viable but nonculturable state. *Appl. Environ. Microbiol.* 64 1736–1742. 9572945 10.1128/aem.64.5.1736-1742.1998 PMC106224

[B23] Borkow G. (2014). *Use of Biocidal Surfaces for Reduction of Healthcare Acquired Infections.* Heidelberg: Springer Press. 10.1007/978-3-319-08057-4

[B24] Braden C. R. (2006). *Salmonella enterica* serotype enteritidis and eggs: a national epidemic in the United States. *Clin. Infect. Dis.* 43 512–517. 10.1086/505973 16838242

[B25] Bridier A. Hammes F. Canette A. Bouchez T. Briandet R. (2015). Fluorescence-based tools for single-cell approaches in food microbiology. *Int. J. Food Microbiol.* 213 2–16. 10.1016/j.ijfoodmicro.2015.07.003 26163933

[B26] Capozzi V. Di Toro M. R. Grieco F. Michelotti V. Salma M. Lamontanara A. (2016). Viable But Not Culturable (VBNC) state of *Brettanomyces bruxellensis* in wine: new insights on molecular basis of VBNC behaviour using a transcriptomic approach. *Food Microbiol.* 59 196–204. 10.1016/j.fm.2016.06.007 27375260

[B27] Cappelier J. M. Besnard V. Roche S. M. Velge P. Federighi M. (2007). Avirulent viable but non culturable cells of *Listeria monocytogenes* need the presence of an embryo to be recovered in egg yolk and regain virulence after recovery. *Vet. Res.* 38 573–583. 10.1051/vetres:2007017 17540159

[B28] Chaisowwong W. Kusumoto A. Hashimoto M. Harada T. Maklon K. Kawamoto K. (2012). Physiological characterization of *Campylobacter jejuni* under cold stresses conditions: its potential for public threat. *J. Vet. Med. Sci.* 74 43–50. 10.1292/jvms.11-0305 21891974

[B29] Chaveerach P. Ter Huurne A. Lipman L. Van Knapen F. (2003). Survival and resuscitation of ten strains of *Campylobacter jejuni* and *Campylobacter coli* under acid conditions. *Appl. Environ. Microbiol.* 69 711–714. 10.1128/AEM.69.1.711-714.2003 12514068 PMC152468

[B30] Clements M. O. Foster S. J. (1998). Starvation recovery of *Staphylococcus aureus* 8325-4. *Microbiology* 144 1755–1763. 10.1099/00221287-144-7-1755 9695909

[B31] Costa K. Bacher G. Allmaier G. Dominguez-Bello M. G. Engstrand L. Falk P. (1999). The morphological transition of *Helicobacter pylori* cells from spiral to coccoid is preceded by a substantial modification of the cell wall. *J. Bacteriol.* 181 3710–3715. 10368145 10.1128/jb.181.12.3710-3715.1999 PMC93848

[B32] Cunningham E. O’Byrne C. Oliver J. D. (2009). Effect of weak acids on *Listeria monocytogenes* survival: evidence for a viable but nonculturable state in response to low pH. *Food Control* 20 1141–1144. 10.1016/j.foodcont.2009.03.005

[B33] Ding T. Suo Y. Xiang Q. Zhao X. Chen S. Ye X. (2016). Significance of viable but nonculturable *Escherichia coli*: induction, detection, and control. *J. Microbiol. Biotechnol.* 10.4014/jmb.1609.09063 [Epub ahead of print]. 27974738

[B34] Dinu L. D. Bach S. (2011). Induction of viable but nonculturable *Escherichia coli* O157: H7 in the phyllosphere of lettuce: a food safety risk factor. *Appl. Environ. Microbiol.* 77 8295–8302. 10.1128/AEM.05020-11 21965401 PMC3233046

[B35] Dinu L.-D. Bach S. (2013). Detection of viable but non-culturable *Escherichia coli* O157: H7 from vegetable samples using quantitative PCR with propidium monoazide and immunological assays. *Food Control* 31 268–273. 10.1016/j.foodcont.2012.10.020

[B36] Du M. Chen J. Zhang X. Li A. Li Y. (2007a). Characterization and resuscitation of viable but nonculturable *Vibrio alginolyticus* VIB283. *Arch. Microbiol.* 188 283–288. 10.1007/s00203-007-0246-5 17492270

[B37] Du M. Chen J. Zhang X. Li A. Li Y. Wang Y. (2007b). Retention of virulence in a viable but nonculturable *Edwardsiella tarda* isolate. *Appl. Environ. Microbiol.* 73 1349–1354. 10.1128/AEM.02243-06 17189433 PMC1828651

[B38] Fakruddin M. Mannan K. S. Andrews S. (2013). Viable but nonculturable bacteria: food safety and public health perspective. *ISRN Microbiol.* 2013:703813 10.1155/2013/703813 PMC3804398 24191231

[B39] Feng Z. Wang Y. An H. Hao Y. Hu X. Liao X. (2016). New insights into the formation of viable but nonculturable *Escherichia coli* O157:H7 induced by high-pressure CO2. *Mbio* 7 e00961–16. 10.1128/mBio.00961-16 27578754 PMC4999544

[B40] Fernández-Delgado M. García-Amado M. A. Contreras M. Incani R. N. Chirinos H. Rojas H. (2015). Survival, induction and resuscitation of *Vibrio cholerae* from the viable but non-culturable state in the Southern Caribbean Sea. *Rev. Inst. Med. Trop. Sao Paulo* 57 21–26. 10.1590/S0036-46652015000100003 PMC4325519 25651322

[B41] Ganesan B. Stuart M. R. Weimer B. C. (2007). Carbohydrate starvation causes a metabolically active but nonculturable state in *Lactococcus lactis*. *Appl. Environ. Microbiol.* 73 2498–2512. 10.1128/AEM.01832-06 17293521 PMC1855592

[B42] Gião M. S. Keevil C. W. (2014). “*Listeria monocytogenes*” can form biofilms in tap water and enter into the viable but non-cultivable state. *Microbiol. Ecol.* 67 603–611. 10.1007/s00248-013-0364-3 24452996

[B43] Gunasekera T. S. Sørensen A. Attfield P. V. Sørensen S. J. Veal D. A. (2002). Inducible gene expression by nonculturable bacteria in milk after pasteurization. *Appl. Environ. Microbiol.* 68 1988–1993. 10.1128/AEM.68.4.1988-1993.2002 11916722 PMC123843

[B44] Gupte A. De Rezende C. Joseph S. (2003). Induction and resuscitation of viable but nonculturable *Salmonella enterica* serovar Typhimurium DT104. *Appl. Environ. Microbiol.* 69 6669–6675. 10.1128/AEM.69.11.6669-6675.2003 14602627 PMC262293

[B45] Habimana O. Nesse L. Møretrø T. Berg K. Heir E. Vestby L. (2014). The persistence of *Salmonella* following desiccation under feed processing environmental conditions: a subject of relevance. *Lett. Appl. Microbiol.* 59 464–470. 10.1111/lam.12308 25046569

[B46] Hsueh P. R. Teng L. J. Tseng S. P. Chang C. F. Wan J. H. Yan J. J. (2004). Ciprofloxacin-resistant *Salmonella enterica* Typhimurium and Choleraesuis from pigs to humans. *Taiwan. Emerg. Infect. Dis.* 10 60–68. 10.3201/eid1001.030171 15078598 PMC3322755

[B47] Hu Y. Coates A. (2012). “Nonmultiplying bacteria are profoundly tolerant to antibiotics,” in *Antibiotic Resistance* eds Anthony R. M. Coates A. (Berlin: Springer Press) 99–119. 10.1007/978-3-642-28951-4_7 23090598

[B48] Hung W.-C. Jane W.-N. Wong H.-C. (2013). Association of a d-alanyl-d-alanine carboxypeptidase gene with the formation of aberrantly shaped cells during the induction of viable but nonculturable *Vibrio parahaemolyticus*. *Appl. Environ. Microbiol.* 79 7305–7312. 10.1128/AEM.01723-13 24056454 PMC3837741

[B49] Imamura D. Mizuno T. Miyoshi S. I Shinoda S. (2015). Stepwise changes in viable but nonculturable *Vibrio cholerae* cells. *Microbiol. Immunol.* 59 305–310. 10.1111/1348-0421.12246 25664673

[B50] Inglis T. J. Sagripanti J.-L. (2006). Environmental factors that affect the survival and persistence of *Burkholderia pseudomallei*. *Appl. Environ. Microbiol.* 72 6865–6875. 10.1128/AEM.01036-06 16980433 PMC1636198

[B51] Jeffreys A. G. Hak K. M. Steffan R. J. Foster J. W. Bej A. K. (1998). Growth, survival and characterization of cspA in *Salmonella enteritidis* following cold shock. *Curr. Microbiol.* 36 29–35. 10.1007/s002849900275 9405743

[B52] Jiang Y. Yan P. Liang J. (2014). Biological changes of *Enterococcus faecalis* in the viable but nonculturable state. *Genet. Mol. Res.* 14 14790–14801. 10.4238/2015.November.18.44 26600540

[B53] Keep N. H. Ward J. M. Cohen-Gonsaud M. Henderson B. (2006a). Wake up! Peptidoglycan lysis and bacterial non-growth states. *Trends Microbiol.* 14 271–276. 10.1016/j.tim.2006.04.003 16675219

[B54] Keep N. H. Ward J. M. Robertson G. Cohen-Gonsaud M. Henderson B. (2006b). Bacterial resuscitation factors: revival of viable but non-culturable bacteria. *Cell. Mol. Life Sci.* 63 2555–2559. 10.1007/s00018-006-6188-2 17013561 PMC11136320

[B55] Kell D. B. Kaprelyants A. S. Weichart D. H. Harwood C. R. Barer M. R. (1998). Viability and activity in reality culturable bacteria: a review and discussion of the practical issues. *Antonie. Van Leeuwenhoek* 73 169–187. 10.1023/A:1000664013047 9717575

[B56] Kogure K. Simidu U. Taga N. (1979). A tentative direct microscopic method for counting living marine bacteria. *Can. J. Microbiol.* 25 415–420. 10.1139/m79-063 378340

[B57] Kramer B. Muranyi P. (2014). Effect of pulsed light on structural and physiological properties of *Listeria innocua* and *Escherichia coli*. *J. Appl. Microbiol.* 116 596–611. 10.1111/jam.12394 24238364

[B58] Kusumoto A. Asakura H. Kawamoto K. (2012). General stress sigma factor RpoS influences time required to enter the viable but non-culturable state in *Salmonella enterica*. *Microbiol. Immunol.* 56 228–237. 10.1111/j.1348-0421.2012.00428.x 22256797

[B59] Lahtinen S. Ahokoski H. Reinikainen J. P. Gueimonde M. Nurmi J. Ouwehand A. C. (2008). Degradation of 16S rRNA and attributes of viability of viable but nonculturable probiotic bacteria. *Lett. Appl. Microbiol.* 46 693–698. 10.1111/j.1472-765X.2008.02374.x 18444975

[B60] Lai C. J. Chen S. Y. Lin I. H. Chang C. H. Wong H. C. (2009). Change of protein profiles in the induction of the viable but nonculturable state of *Vibrio parahaemolyticus*. *Int. J. Food Microbiol.* 135 118–124. 10.1016/j.ijfoodmicro.2009.08.023 19735955

[B61] Lázaro B. Cárcamo J. Audícana A. Perales I. Fernández-Astorga A. (1999). Viability and DNA maintenance in nonculturable spiral *Campylobacter jejuni* cells after long-term exposure to low temperatures. *Appl. Environ. Microbiol.* 65 4677–4681. 10508106 10.1128/aem.65.10.4677-4681.1999 PMC91624

[B62] Léonard L. Bouarab Chibane L. Ouled Bouhedda B. Degraeve P. Oulahal N. (2016). Recent advances on multi-parameter flow cytometry to characterize antimicrobial treatments. *Front. Microbiol.* 7:1225 10.3389/fmicb.2016.01225 PMC4976717 27551279

[B63] Li J. Ahn J. Liu D. Chen S. Ye X. Ding T. (2016). Evaluation of ultrasound-induced damage to *Escherichia coli* and *Staphylococcus aureus* by flow cytometry and transmission electron microscopy. *Appl. Environ. Microbiol.* 82 1828–1837. 10.1128/AEM.03080-15 26746712 PMC4784048

[B64] Li L. Mendis N. Trigui H. Oliver J. D. Faucher S. P. (2014). The importance of the viable but non-culturable state in human bacterial pathogens. *Front. Microbiol.* 5:258 10.3389/fmicb.2014.00258 PMC4040921 24917854

[B65] Lin Y.-W. Li D. Gu A. Z. Zeng S. Y. He M. (2016). Bacterial regrowth in water reclamation and distribution systems revealed by viable bacterial detection assays. *Chemosphere* 144 2165–2174. 10.1016/j.chemosphere.2015.10.071 26595310

[B66] Linder K. Oliver J. D. (1989). Membrane fatty acid and virulence changes in the viable but nonculturable state of *Vibrio vulnificus*. *Appl. Environ. Microbiol.* 55 2837–2842. 2696428 10.1128/aem.55.11.2837-2842.1989 PMC203178

[B67] Liu J. Rong Z. Lin L. Peters B. M. Bing L. Lin C. W. (2016). Viable but non-culturable state and toxin gene expression of enterohemorrhagic *Escherichia coli* O157 under cryopreservation. *Res. Microbiol.* 168 188–193. 10.1016/j.resmic.2016.11.002 27884785

[B68] Lleo M. Bonato B. Tafi M. Signoretto C. Boaretti M. Canepari P. (2001). Resuscitation rate in different enterococcal species in the viable but non-culturable state. *J. Appl. Microbiol.* 91 1095–1102. 10.1046/j.1365-2672.2001.01476.x 11851818

[B69] Lothigius A. Sjoling A. Svennerholm A. M. Bölin I. (2010). Survival and gene expression of enterotoxigenic *Escherichia coli* during long-term incubation in sea water and freshwater. *J. Appl. Microbiol.* 108 1441–1449. 10.1111/j.1365-2672.2009.04548.x 19804537

[B70] Magajna B. A. Schraft H. Schraft H. (2015). *Campylobacter jejuni* biofilm cells become viable but non-culturable (VBNC) in low nutrient conditions at 4°C more quickly than their planktonic counterparts. *Food Control* 50 45–50. 10.1016/j.foodcont.2014.08.022

[B71] Magnusson L. U. Farewell A. Nyström T. (2005). ppGpp: a global regulator in *Escherichia coli*. *Trends Microbiol.* 13 236–242. 10.1016/j.tim.2005.03.008 15866041

[B72] Makino S.-I. Kii T. Asakura H. Shirahata T. Ikeda T. Takeshi K. (2000). Does Enterohemorrhagic *Escherichia coli* O157: H7 enter the viable but nonculturable state in salted salmon roe? *Appl. Environ. Microbiol.* 66 5536–5539. 10.1128/AEM.66.12.5536-5539.2000 11097946 PMC92500

[B73] Masmoudi S. Denis M. Maalej S. (2010). Inactivation of the gene katA or sodA affects the transient entry into the viable but non-culturable response of *Staphylococcus aureus* in natural seawater at low temperature. *Mar. Pollut. Bull.* 60 2209–2214. 10.1016/j.marpolbul.2010.08.017 20833402

[B74] Mathur H. Rea M. Fallico V. Cotter P. Hill C. Ross P. (2016). Flow cytometry as a tool to study the effects of bacteriocins on prokaryotic and eukaryotic cells. *J. Mol. Biomark. Diagn.* S8:013 10.4172/2155-9929.S8-013

[B75] Meyer B. (2006). Does microbial resistance to biocides create a hazard to food hygiene? *Int. J. Food. Microbiol.* 112 275–279. 10.1016/j.ijfoodmicro.2006.04.012 16769146

[B76] Moorhead S. Griffiths M. (2011). Expression and characterization of cell-signalling molecules in *Campylobacter jejuni*. *J. Appl. Microbiol.* 110 786–800. 10.1111/j.1365-2672.2010.04934.x 21205102

[B77] Morishige Y. Fujimori K. Amano F. (2013). Differential resuscitative effect of pyruvate and its analogues on VBNC (Viable But Non-Culturable) *Salmonella*. *Microbes Environ.* 28 180–186. 10.1264/jsme2.ME12174 23595023 PMC4070669

[B78] Mukamolova G. V. Kaprelyants A. S. Kell D. B. Young M. (2003). Adoption of the transiently non-culturable state–a bacterial survival strategy? *Adv. Microb. Physiol.* 47 65–129. 10.1016/S0065-2911(03)47002-1 14560663

[B79] Mukamolova G. V. Kaprelyants A. S. Young D. I. Young M. Kell D. B. (1998). A bacterial cytokine. *Proc. Natl. Acad. Sci. U.S.A.* 95 8916–8921. 10.1073/pnas.95.15.8916 9671779 PMC21177

[B80] Nicolò M. S. Gioffrè A. Carnazza S. Platania G. Silvestro I. D. Guglielmino S. P. (2010). Viable but nonculturable state of foodborne pathogens in grapefruit juice: a study of laboratory. *Foodborne Pathog. Dis.* 8 11–17. 10.1089/fpd.2009.0491 20932087

[B81] Nicolò M. S. Guglielmino S. P. P. (2012). “Viable but nonculturable bacteria in food,” in *Public Health–Methodology, Environmental and Systems Issues* ed. Maddock J. (Rjeka: InTech) 189–216. 10.5772/38118

[B82] Nocker A. Camper A. K. (2009). Novel approaches toward preferential detection of viable cells using nucleic acid amplification techniques. *FEMS. Microbiol. Lett.* 291 137–142. 10.1111/j.1574-6968.2008.01429.x 19054073

[B83] Nowakowska J. Oliver J. D. (2013). Resistance to environmental stresses by *Vibrio vulnificus* in the viable but nonculturable state. *FEMS. Microbiol. Ecol.* 84 213–222. 10.1111/1574-6941.12052 23228034

[B84] Nyström T. (2003). Nonculturable bacteria: programmed survival forms or cells at death’s door? *Bioessays.* 25 204–211. 10.1002/bies.10233 12596224

[B85] Oh E. McMullen L. Jeon B. (2015). Impact of oxidative stress defense on bacterial survival and morphological change in *Campylobacter jejuni* under aerobic conditions. *Front. Microbiol* 6:295 10.3389/fmicb.2015.00295 PMC4392298 25914692

[B86] Oliver J. D. (2005). The viable but nonculturable state in bacteria. *J. Microbiol.* 43 93–100. 15765062

[B87] Oliver J. D. (2010). Recent findings on the viable but nonculturable state in pathogenic bacteria. *FEMS. Microbiol. Rev.* 34 415–425. 10.1111/j.1574-6976.2009.00200.x 20059548

[B88] Oliver J. D. Bockian R. (1995). In vivo resuscitation, and virulence towards mice, of viable but nonculturable cells of *Vibrio vulnificus*. *Appl. Environ. Microbiol.* 61 2620–2623. 10.3109/02713680903477824 7618873 PMC167533

[B89] Orman M. A. Brynildsen M. P. (2013). Establishment of a method to rapidly assay bacterial persister metabolism. *Antimicrob. Agents Chemother.* 57 4398–4409. 10.1128/AAC.00372-13 23817376 PMC3754326

[B90] Paniel N. Baudart J. Hayat A. Barthelmebs L. (2013). Aptasensor and genosensor methods for detection of microbes in real world samples. *Methods* 64 229–240. 10.1016/j.ymeth.2013.07.001 23872322

[B91] Panutdaporn N. Kawamoto K. Asakura H. Makino S. I. (2006). Resuscitation of the viable but non-culturable state of *Salmonella enterica* serovar Oranienburg by recombinant resuscitation-promoting factor derived from *Salmonella Typhi*murium strain LT2. *Int. J. Food Microbiol.* 106 241–247. 10.1016/j.ijfoodmicro.2005.06.022 16213054

[B92] Pasquaroli S. Citterio B. Cesare A. D. Amiri M. Manti A. Vuotto C. (2014). Role of daptomycin in the induction and persistence of the viable but non-culturable state of *Staphylococcus aureus* biofilms. *Pathogens* 3 759–768. 10.3390/pathogens3030759 25438023 PMC4243440

[B93] Pasquaroli S. Zandri G. Vignaroli C. Vuotto C. Donelli G. Biavasco F. (2013). Antibiotic pressure can induce the viable but non-culturable state in *Staphylococcus aureus* growing in biofilms. *J. Antimicrob. Chemother.* 68 1812–1817. 10.1093/jac/dkt086 23515246

[B94] Patrone V. Campana R. Vallorani L. Dominici S. Federici S. Casadei L. (2013). CadF expression in *Campylobacter jejuni* strains incubated under low-temperature water microcosm conditions which induce the viable but non-culturable (VBNC) state. *Antoni. Van Leeuwenhoek* 103 979–988. 10.1007/s10482-013-9877-5 23314927

[B95] Pawlowski D. R. Metzger D. J. Raslawsky A. Howlett A. Siebert G. Karalus R. J. (2011). Entry of Yersinia pestis into the viable but nonculturable state in a low-temperature tap water microcosm. *PLoS ONE* 6:e17585 10.1371/journal.pone.0017585 PMC3059211 21436885

[B96] Peneau S. Chassaing D. Carpentier B. (2007). First evidence of division and accumulation of viable but nonculturable *Pseudomonas fluorescens* cells on surfaces subjected to conditions encountered at meat processing premises. *Appl. Environ. Microbiol.* 73 2839–2846. 10.1128/AEM.02267-06 17337551 PMC1892859

[B97] Pianetti A. Battistelli M. Barbieri F. Bruscolini F. Falcieri E. Manti A. (2012). Changes in adhesion ability of Aeromonas hydrophila during long exposure to salt stress conditions. *J. Appl. Microbiol.* 113 974–982. 10.1111/j.1365-2672.2012.05399.x 22805151

[B98] Pinto D. Almeida V. Almeida Santos M. Chambel L. (2011). Resuscitation of *Escherichia coli* VBNC cells depends on a variety of environmental or chemical stimuli. *J. Appl. Microbiol.* 110 1601–1611. 10.1111/j.1365-2672.2011.05016.x 21447017

[B99] Pinto D. Santos M. A. Chambel L. (2015). Thirty years of viable but nonculturable state research: unsolved molecular mechanisms. *Crit. Rev. Microbiol.* 41 61–76. 10.3109/1040841X.2013.794127 23848175

[B100] Pinto D. Sãojosé C. Santos M. A. Chambel L. (2013). Characterization of two resuscitation promoting factors of *Listeria monocytogenes*. *Microbiology* 159 1390–1401. 10.1099/mic.0.067850-0 23676438

[B101] Pommepuy M. Butin M. Derrien A. Gourmelon M. Colwell R. Cormier M. (1996). Retention of enteropathogenicity by viable but nonculturable *Escherichia coli* exposed to seawater and sunlight. *Appl. Environ. Microbiol.* 62 4621–4626. 8953732 10.1128/aem.62.12.4621-4626.1996 PMC168287

[B102] Radomski N. Moilleron R. Lucas F. S. Falkinham I. I. I. J. (2010). “Challenges in environmental monitoring of pathogens: case study in Mycobacterium avium,” in *Current Research, Technology and Education Topics in Applied Microbiology and Microbial Biotechnology* ed. Méndez-Vilas A. (Extremadura: Formatex Research Center) 1551–1561.

[B103] Rahman F. Noor R. (2012). Prevalence of pathogenic bacteria in common salad vegetables. *Bangl. J. Bot.* 41 159–162.

[B104] Rahman I. Shahamat M. Chowdhury M. Colwell R. (1996). Potential virulence of viable but nonculturable *Shigella dysenteriae* type 1. *Appl. Environ. Microbiol.* 62 115–120. 8572688 10.1128/aem.62.1.115-120.1996 PMC167780

[B105] Rahman I. Shahamat M. Kirchman P. Russek-Cohen E. Colwell R. (1994). Methionine uptake and cytopathogenicity of viable but nonculturable *Shigella dysenteriae* type 1. *Appl. Environ. Microbiol.* 60 3573–3578. 7986035 10.1128/aem.60.10.3573-3578.1994 PMC201857

[B106] Ramamurthy T. Ghosh A. Pazhani G. P. Shinoda S. (2014). Current Perspectives on Viable but Non-Culturable (VBNC) Pathogenic Bacteria. *Front. Public Health* 2:103 10.3389/fpubh.2014.00103 PMC4116801 25133139

[B107] Rao N. V. Shashidhar R. Bandekar J. R. (2014). Induction, resuscitation and quantitative real-time polymerase chain reaction analyses of viable but nonculturable *Vibrio vulnificus* in artificial sea water. *World J. Microbiol. Biotechnol.* 30 2205–2212. 10.1007/s11274-014-1640-1 24696138

[B108] Ravel J. Knight I. T. Monahan C. E. Hill R. T. Colwell R. R. (1995). Temperature-induced recovery of *Vibrio cholerae* from the viable but nonculturable state: growth or resuscitation? *Microbiology* 141 377–383. 10.1099/13500872-141-2-377 7704268

[B109] Roszak D. Grimes D. Colwell R. (1984). Viable but nonrecoverable stage of *Salmonella enteritidis* in aquatic systems. *Can. J. Microbiol.* 30 334–338. 10.1139/m84-049 6372975

[B110] Rowan N. J. (2004). Viable but non-culturable forms of food and waterborne bacteria: quo vadis? *Trends Food Sci. Technol.* 15 462–467. 10.1016/j.tifs.2004.02.009

[B111] Rowan N. J. Valdramidis V. P. Gomez-Lopez V. M. (2015). A review of quantitative methods to describe efficacy of pulsed light generated inactivation data that embraces the occurrence of viable but non culturable state microorganisms. *Trends Food Sci. Technol.* 44 79–92. 10.1016/j.tifs.2015.03.006

[B112] Sachidanandham R. Gin K. (2009). A dormancy state in nonspore-forming bacteria. *Appl. Microbiol. Biotechnol.* 81 927–941. 10.1007/s00253-008-1712-y 18815783 PMC7419491

[B113] Scheutz F. Nielsen E. M. Frimodt-Moller J. Boisen N. Morabito S. Tozzoli R. (2011). Characteristics of the enteroaggregative Shiga toxin/verotoxin-producing *Escherichia coli* O104: H4 strain causing the outbreak of haemolytic uraemic syndrome in Germany, May to June 2011. *Euro Surveill.* 16 19889. 10.2807/ese.16.24.19889-en 21699770

[B114] Senoh M. Ghosh-Banerjee J. Ramamurthy T. Hamabata T. Kurakawa T. Takeda M. (2010). Conversion of viable but nonculturable *Vibrio cholerae* to the culturable state by co-culture with eukaryotic cells. *Microbiol. Immunol.* 54 502–507. 10.1111/j.1348-0421.2010.00245.x 20840148

[B115] Sheridan G. E. C. Masters C. I. Shallcross J. A. Mackey B. M. (1998). Detection of mRNA by reverse transcription-PCR as an indicator of viability in *Escherichia coli* cells. *Appl. Environ. Microbiol.* 64 1313–1318. 9546166 10.1128/aem.64.4.1313-1318.1998 PMC106147

[B116] Signoretto C. del Mar Lleo M. Canepari P. (2002). Modification of the peptidoglycan of *Escherichia coli* in the viable but nonculturable state. *Curr. Microbiol.* 44 125–131. 10.1007/s00284-001-0062-0 11815857

[B117] Signoretto C. Lleò M. M. Tafi M. C. Canepari P. (2000). Cell wall chemical composition of *Enterococcus faecalis* in the viable but nonculturable state. *Appl. Environ. Microbiol.* 66 1953–1959. 10.1128/AEM.66.5.1953-1959.2000 10788366 PMC101439

[B118] Smith B. Oliver J. D. (2006). In situ and in vitro gene expression by *Vibrio vulnificus* during entry into, persistence within, and resuscitation from the viable but nonculturable state. *Appl. Environ. Microbiol.* 72 1445–1451. 10.1128/AEM.72.2.1445-1451.2006 16461698 PMC1392903

[B119] Su C. P. Jane W. N. Wong H. C. (2013). Changes of ultrastructure and stress tolerance of *Vibrio parahaemolyticus* upon entering viable but nonculturable state. *Int. J. Food. Microbiol.* 160 360–366. 10.1016/j.ijfoodmicro.2012.11.012 23290246

[B120] Trevors J. Elsas J. Bej A. (2012). The molecularly crowded cytoplasm of bacterial cells: dividing cells contrasted with viable but non-culturable (VBNC) bacterial cells. *Curr. Issues Mol. Biol.* 15 1–6. 22513407

[B121] Trevors J. T. (2011). Viable but non-culturable (VBNC) bacteria: gene expression in planktonic and biofilm cells. *J. Microbiol. Methods* 86 266 10.1016/j.mimet.2011.04.018 21616099

[B122] Trinh N. T. T. Dumas E. Thanh M. L. Degraeve P. Amara C. B. Gharsallaoui A. (2015). Effect of a Vietnamese Cinnamomum cassia essential oil and its major component trans-cinnamaldehyde on the cell viability, membrane integrity, membrane fluidity, and proton motive force of *Listeria innocua*. *Can. J. Microbiol.* 61 263–271. 10.1139/cjm-2014-0481 25728340

[B123] Trudeau K. Vu K. D. Shareck F. Lacroix M. (2012). Capillary electrophoresis separation of protein composition of γ-irradiated food pathogens *Listeria monocytogenes* and *Staphylococcus aureus*. *PLoS ONE* 7:e32488 10.1371/journal.pone.0032488 PMC3299667 22427846

[B124] Whitesides M. D. Oliver J. D. (1997). Resuscitation of *Vibrio vulnificus* from the viable but nonculturable state. *Appl. Environ. Microbiol.* 63 1002–1005. 16535534 10.1128/aem.63.3.1002-1005.1997 PMC1389128

[B125] Wong H. C. Wang P. (2004). Induction of viable but nonculturable state in *Vibrio parahaemolyticus* and its susceptibility to environmental stresses. *J. Appl. Microbiol.* 96 359–366. 10.1046/j.1365-2672.2004.02166.x 14723697

[B126] Xu H. S. Roberts N. Singleton F. L. Attwell R. W. Grimes D. J. Colwell R. R. (1982). Survival and viability of nonculturable *Escherichia coli* and *Vibrio cholerae* in the estuarine and marine environment. *Microb. Ecol.* 8 313–323. 10.1007/BF02010671 24226049

[B127] Xuan X. T. Ding T. Li J. Ahn J. H. Zhao Y. Chen S. G. (2017). Estimation of growth parameters of *Listeria monocytogenes* after sublethal heat and slightly acidic electrolyzed water (SAEW) treatment. *Food Control* 71 17–25. 10.1016/j.foodcont.2016.06.018

[B128] Yaron S. Matthews K. (2002). A reverse transcriptase-polymerase chain reaction assay for detection of viable *Escherichia coli* O157: H7: investigation of specific target genes. *J. Appl. Microbiol.* 92 633–640. 10.1046/j.1365-2672.2002.01563.x 11966903

[B129] Zeng B. Zhao G. Cao X. Yang Z. Wang C. Hou L. (2012). Formation and resuscitation of viable but nonculturable *Salmonella typhi*. *BioMed. Res. Int.* 2013:907170 10.1155/2013/907170 PMC3591152 23509799

[B130] Zhang S. Ye C. Lin H. Lv L. Yu X. (2015). UV disinfection induces a VBNC state in *Escherichia coli* and *Pseudomonas aeruginosa*. *Environ. Sci. Technol.* 49 1721–1728. 10.1021/es505211e 25584685

[B131] Zhao F. Bi X. Hao Y. Liao X. (2013). Induction of viable but nonculturable *Escherichia coli* O157: H7 by high pressure CO2 and its characteristics. *PLoS ONE* 8:e62388 10.1371/journal.pone.0062388 PMC3633907 23626816

[B132] Zhao X. Lin C. W. Wang J. Oh D. H. (2014). Advances in rapid detection methods for foodborne pathogens. *J. Microbiol. Biotechnol.* 24 297–312. 10.4014/jmb.1310.10013 24375418

[B133] Zhao X. Wang J. Forghani F. Park J. H. Park M. S. Seo K. H. (2013). Rapid detection of viable *Escherichia coli* O157 by coupling propidium monoazide with loop-mediated isothermal amplification. *J. Microbiol. Biotechnol.* 23 1708–1716. 10.4014/jmb.1306.06003 24002453

[B134] Zhao X. Wei C. Zhong J. Jin S. (2016). Research advance in rapid detection of foodborne *Staphylococcus aureus*. *Biotechnol. Biotechnol. Equip.* 30 1–7. 10.1080/13102818.2016.1209433

[B135] Zhong Q. Tian J. Wang B. Wang L. (2016). PMA based real-time fluorescent LAMP for detection of *Vibrio parahaemolyticus* in viable but nonculturable state. *Food Control* 63 230–238. 10.1016/j.foodcont.2015.11.043

[B136] Ziprin R. L. Droleskey R. E. Hume M. E. Harvey R. B. (2003). Failure of viable nonculturable *Campylobacter jejuni* to colonize the cecum of newly hatched leghorn chicks. *Avian. Dis.* 47 753–758. 10.1637/7015 14562908

